# What should global mental health do about violent extremism?

**DOI:** 10.1017/gmh.2019.12

**Published:** 2019-07-16

**Authors:** S. Weine, S. Kansal

**Affiliations:** College of Medicine, University of Illinois, Chicago, IL, USA

**Keywords:** Violent extremism

## Abstract

To prevent radicalization to violence and to rehabilitate returned foreign terrorist fighters, new programs which go by the name of ‘preventing and countering violent extremism’ are being implemented globally, including in low- and middle-income countries. In some of these countries, global mental health strategies are also being implemented so as to deliver mental health care or psychosocial support to individuals and populations in need. This commentary addresses what global mental health should considering doing about violent extremism. Global mental health should be open to addressing the challenges of violent extremism but should do so based upon existing mental health and public health values, practices, and evidence. Global mental health could help by critically appraising preventing and countering violent extremism practices and by working with multidisciplinary stakeholders to develop new evidence-based and best practice models that are rooted in civil society ownership, community collaboration, broader prevention programing, and non-securitized approaches.

## Introduction

Global mental health (GMH) strategies are being implemented in many low- and middle-income countries so as to deliver mental health care or psychosocial support to those in need who have not had access to such services. At the same time, to address current public safety priorities, such as preventing radicalization to violence and rehabilitating returned foreign terrorist fighters, new programs which go by the name of ‘preventing and countering violent extremism’ (P/CVE) are also being implemented in some of those same countries.

P/CVE is often organized by national security agencies or law enforcement, but is unique in how it enlists other government agencies and civil society, including mental health professionals and educators, in efforts to diminish violent extremism and terrorism. For example, law enforcement officers may be expected to refer individuals considered at risk for violence to mental health professionals or clergy. Teachers may be expected to identify and refer their students who exhibit violent radical ideas or threaten violence. Mental health professionals may be expected to assess whether a person is at-risk for committing an act of violence and to provide treatment which prevents violence. However, to date these new expectations are not yet supported by adequate scientific evidence and best practices.

In this commentary, we follow the lead of international development organizations by including ‘preventing violent extremism’ so as to delineate an approach which is intended to be less securitized, more community-oriented, and more focused on preventing a broad range of negative outcomes, then its more security driven predecessor, ‘countering violent extremism’.

Even with this reframing, questions are still being asked as to whether low- and middle-income countries should commit precious mental health resources to P/CVE, given the scope of the need for mental health care, the limitations of mental health resources, and concerns regarding the lack of evidence of effectiveness, and the risks of over-securitization posed by P/CVE.

The purpose of this paper is to better understand whether, and possibly how, GMH should be concerned with violent extremism and interact with P/CVE. We argue that GMH should be open to addressing the challenge of P/CVE in low- and middle-income countries, but that even this reframing of P/CVE is not enough to overcome the limitations and burdens associated with these programs. Rather, even further reframing is necessary, to which GMH could possibly contribute.

## Global mental health

The field of GMH aims to address mental health challenges amidst socio-economic adversity and social suffering. It addresses specific challenges such as integrating mental health screening, diagnosis, and care into primary care, providing affordable and effective community-based care, strengthening mental health training of all healthcare personnel, and promoting the value of mental health. The GMH focus is largely on low- and middle-income countries, where these difficulties are often highly prevalent and where existing mental health resources and infrastructure are often low (Becker & Kleinman, 2013).

Indeed, GMH research initiatives, like the Grand Challenges in Global Mental Health Initiative and the Movement for Global Mental Health have led to better characterization of mental health needs in low- and middle-income countries, more effective intervention approaches that can work in these countries, reduced stigma regarding care of the mentally ill, development of mental health prevention and promotion strategies, and an increased mental health research capacity (Collins *et al*., 2011). Particular areas of ongoing concern involve the physical and mental health care of children, defining the earliest identifiable clinical stages of illness, and reducing the long-term negative impact of poverty and trauma on brain development.

Task sharing, one of the key strategies for GMH, is used for overall mental health promotion and to address gaps in mental health services in the face of inadequate mental health resources. It involves training laypersons and mid-level professionals so they can help provide mental health services. Task sharing has been used to address common mental disorders such as depression and anxiety, the impact of social adversity, as well as medical problems such as HIV/AIDS (Petersen *et al*., 2011; Ledikwe *et al*., 2013). For example, task sharing involving lay counselors helped to reduce stigma regarding HIV and to increase the utilization of medical services (Ledikwe *et al*., 2013).

## Preventing and countering violent extremism

P/CVE emphasizes working collaboratively with communities so as to empower them to address the underlying causes of violent extremism and to prevent terrorist attacks before they occur. Unlike counter-terrorism, which is driven primarily by law enforcement, P/CVE can involve a wide range of government agencies and civil society actors, including mental health professionals, educators, youth advocates, clergy, community organizers, public health professionals, and job coaches, among others. It can incorporate promoting social cohesion and building community resilience (Weine & Ahmed, 2012; Ellis & Abdi, 2017). CVE and PVE as policy frameworks were articulated in documents by the White House (White House, 2011) and the U.N. (Report of the Secretary-General, 2015), and have been subject to several recent reviews (Romaniuk, 2015; GAO, 2017; Rosand *et al*., 2018).

In March 2015, the White House Summit on CVE devoted one entire day to global CVE, and foreign ministers and civil society participants from 60 nations attended. President Obama spoke of creating paths for ‘opportunity, justice, and dignity’ (Obama, 2015) so that one is not tempted to throw their life away to violent acts. He stated, ‘our campaign to prevent people around the world being radicalized to violence is ultimately a battle for hearts and minds’ (Obama, 2015). The foreign ministers signed a statement which called for ‘community-based strategies’ and this mobilized resources and support for the development of new CVE initiatives in many countries including low- and middle-income countries.

Despite the change in White House administration, P/CVE remains active in global spaces (Iroegbu, 2017; Rosand, 2017). P/CVE activities are highly embedded in international organizations, law enforcement agencies, donor governments, development agencies, and non-governmental organizations. Over the past several years, P/CVE initiatives have spread to many low- and middle-income countries, through the U.S. State Department, the European Union, the Global Counterterrorism Forum, the Hedayah Center, the Strong City Network, the Global Community and Engagement Fund, and more recently through development agencies such as the U.N. Development Program and the World Bank (Department of State, 2016; UNDP, 2016; Global Counterterrorism Forum, 2017; Hedayah Center, 2017; Regional Capacity, 2017; Rosand, 2017). Consequently, P/CVE programing is making its way into some of the same low- and middle-income countries as the GMH movement, enabling potential collaboration.

The spectrum of P/CVE activities increasingly follows the public health model of primary, secondary, and tertiary prevention (Bhui *et al*., 2012; Weine *et al*., 2016). Primary prevention targets the whole community through activities that aim to shift cultural norms, enhance social cohesion, or improve access to services. Secondary prevention focuses on identifying and providing early intervention services for those presenting at a higher risk to act violently. Tertiary prevention activities focus on rehabilitation and reintegration of those who have already committed an act of violence or some crime related to terrorism (Dean & Kessels, 2018).

Mental health professionals can play key roles at each of these levels. They can develop, implement, and/or evaluate prevention, intervention, and rehabilitation and reintegration programs. Mental health professionals can assess and possibly treat persons at risk for violence (Weine *et al*., 2017). Their standard professional practices for assessment, diagnosis, and therapeutic management can be valuable, even more so if supplemented by training in specialized practices such as threat assessment (Meloy & Hoffman, 2014). Current practices in P/CVE emphasize developing multi-agency/multi-disciplinary referral mechanisms to include violent extremism alongside multiple other issues addressed, such as other types of violence, other crises, and suicide (Kozmelj, 2017).

The implementation of P/CVE has not been without challenges, including in the United States and UK. P/CVE has yet to establish an evidence-base of effective programs, or agreed upon outcome metrics, despite substantial funding (Holmer *et al*., 2018). In the United States, many community advocates and civil libertarians have opposed CVE as a hurtful program of government surveillance on Muslim communities (Brennan Center for Justice, 2017). In the UK, where the government's Prevent program (which includes Channel) entails reporting responsibilities for National Health Service physicians, some psychiatrists have challenged the policies on the basis of the harm they could do to the patient–physician relationship and the lack of an adequate evidence base (Hurlow *et al*., 2016; James & Hurlow, 2016; McGarry, 2016; Summerfield, 2016; Dom *et al*., 2018).

On the other hand, some local pilot initiatives that are being currently implemented in the United States and abroad deliberately move away from P/CVE framing. Rather, they focus on community-led approaches to building individual and community resilience, promoting youth development, improving access to mental health services and reducing involvement in all forms of violence (Rosand *et al*., 2018). These appear to be getting better responses from communities and professionals and could indicate more promising future directions. There is also a growing body of research on the factors that contribute to violent extremism, such as deficits in local governance (Rosand *et al*., 2018), which could help increase the likelihood that future programs are designed to address the identified drivers of violent extremism.

## Comparing global mental health and preventing and countering violent extremism

Despite the intent for civil society to play a leading role in P/CVE programs, P/CVE has often been led by law enforcement and governmental agencies. In the United States, P/CVE has been more based upon criminal justice practice, than on public health or mental health, although this has been changing in recent years (Weine *et al*., 2016). Like in GMH, the hope for P/CVE globally has been to involve a broad spectrum of civil society actors, such as clergy, educators, youth advocates, and mental health professionals.

Unlike GMH, the key documents in P/CVE are more focused on articulating a broad policy or practice agenda then on implementable prevention program packages. Far more research has been conducted in GMH than P/CVE, including program evaluation, in part because these programs have been around longer, and because the scientific approaches to evaluating them are more developed.

In the United States, P/CVE has had difficulty establishing community buy-in for its programing, and is opposed by some advocates who assert that P/CVE is inherently stigmatizing and is surveillance by other means (Brennan Center for Justice, 2017). GMH has been much more successful in establishing community connections (Patel *et al*., 2011), whereas in the United States, P/CVE struggles to make community inroads.

P/CVE may able to attract resources that GMH cannot typically access, and yet, some civil society organizations in high-income countries have decided not to seek or accept government funding (Wang, 2017). More so than in Western countries, P/CVE in low- and middle-income countries in the Global South tends to be more focused on community-based primary prevention activities – often led by internationally-funded civil society groups – that don't involve the police and aren't dealing with secondary prevention. This is likely due to greater lack of trust between police (and government more broadly) and communities, more limited capacities of government, and fewer mental health and other resources required to develop and sustain secondary prevention programs (Rosand & Winterbotham, 2018).

With these distinctions in mind, we want to consider a number of possible ways in which GMH and P/CVE may interact, both positively and negatively.

## How can preventing and countering violent extremism and global mental health positively converge?

Positive convergence occurs when strengths and practices combine in ways that contribute to more positive outcomes. Potential areas of positive convergence between P/CVE and GMH may involve increasing access to mental health resources, preventing violence, combating stigma, and leveraging successful GMH strategies.

Both GMH and P/CVE are concerned with *providing mental health resources* to those who need them but lack access. P/CVE is focused on reaching those communities/individuals who are most at risk of becoming radicalized to violence, but need not have already committed crimes or become violent. GMH has focused on bringing mental health resources to persons in need and who lack resources. P/CVE initiatives could make mental health resources available and accessible to sub-populations of interest, such as through community-based referral mechanisms. This could include utilizing task sharing and/or clinical approaches.

Both GMH and P/CVE are concerned with *violence prevention*. GMH has been more focused on interpersonal violence, including child maltreatment, youth violence, intimate partner and sexual violence, and elder abuse as well as the impact of war and conflict on civilians, especially refugees, internally displaced persons, and asylum seekers (de Jong, 2006). P/CVE is often focused on ideologically inspired violence; however, there is ongoing debate as to whether this is too narrow (Weine *et al*., 2016). Both GMH and P/CVE could widen the focus to targeted violence (violence where a known or knowable attacker selects a specific target prior to attack), hate crimes, and interpersonal violence, which would align with the violence prevention agenda of public health (Christoffel & Gallagher, 2006). As mentioned earlier, in the public health field of injury prevention, there is a growing body of violence prevention theory, evidence, and practice models, which both P/CVE and GMH could leverage (Bhui *et al*., 2012; Weine *et al*., 2016).

Both P/CVE and GMH share concerns with combating stigma. GMH has had an ongoing commitment to diminishing the stigma toward people with mental illness and toward seeking treatment. P/CVE has been criticized for causing stigmatization by singling out communities or individuals for heightened risk of violent extremism who do not consider themselves to be at risk. In response, P/CVE initiatives have taken steps to avoid focusing on single faith communities, and to instead focus on any persons or communities where there is demonstrable heightened risk. They have also strived to address not only violent extremism but also targeted violence and hate crimes. P/CVE needs to take further steps to challenge stigma in impacted populations through building knowledge, changing attitudes, improving practices, and strengthening community collaboration.

P/CVE could leverage successful GMH strategies especially regarding partnership, engagement, and task sharing. GMH has had far more experience and success in forming sustainable partnerships than P/CVE. P/CVE struggles to overcome a fundamental challenge: because P/CVE is often seen as a political and security issue, it is difficult to build partnerships with non-law enforcement professionals, including with mental health. GMH has had far more experience with building and evaluating programs, including how to engage hard to reach populations, how to develop partnerships, how to promote program sustainability, how to build evidence for effective interventions, and how to stand united with survivors, family members, formers, and providers.

P/CVE could embrace task sharing approaches to mental health, particularly in communities where there are concerns about risk for radicalization to violence. These programs do not necessarily have to be focused on radicalization; they could be what is referred to as P/CVE relevant, without being P/CVE specific. In other words, instead of emphasizing the uniqueness of P/CVE, they could bring P/CVE closer to existing GMH concerns regarding social determinants, psychosocial services, and the everyday needs of families, schools, and communities (e.g. strengthening social cohesion).

## What are possible points of negative convergence of countering violent extremism and global mental health?

Negative convergence occurs when risks and practices combine in ways that contribute to more negative outcomes. Potential areas of negative convergence between P/CVE and GMH may involve failure to launch, rush to judgment, exacerbating stigma, over-securitized relationship, and lack of capacity.

Failure to launch can occur when mental health professionals, including those in the GMH community, simply do not acknowledge violent extremism or hate-motivated violence as any kind of priority or relevant problem for them to address. They may see it as today's fashion for getting funds, and not as connecting with their core obligations and priorities. For example, as noted above, in the UK, several questioned whether mental health professionals should be involved in P/CVE, which they regarded as ‘political' and potentially compromising professional ethics (McGarry, 2016). With so much on GMH's plate already, violent extremism can easily be pushed aside. Yet, although violent extremist acts may be relatively infrequent, the effects are devastating, and should not be neglected. It is also worth noting that if GMH professionals do not get specialized training in P/CVE, then there is no real possibility of positive convergence between the fields.

*Rush to judgment*, on the other hand, can occur around prematurely deciding upon the role of mental illness as a ‘cause' of violent extremism, or over-relying upon unsound and discredited theories, such as radicalization theory. This could lead to activities that are not adequately justified or are mis-focused, or even discriminatory. Indeed, research has not yet demonstrated a clear association between mental health and violent extremism (Corner & Gill, 2015). Overreaching can lead to pushback.

*Exacerbating stigma* is an oft mentioned issue, and refers to how associating individuals, families, or communities with terrorism can mark them with disgrace and infamy.

Mental illness and mental health treatment are also highly stigmatized in low- and middle-income countries (Weine *et al*., 2017). Thus, highlighting an additional association between mental illness and violent extremism, or violence of any kind, could further exacerbate stigma for individuals, families, or communities, and in turn work against whatever progress has been made in getting people in low- and middle-income countries to accept mental health services.

*Over-securitized relationships* refer to concerns that involvement in P/CVE could lead to damaging the trust held by patients and their families and communities in practitioners, service organization, or the mental health professions. The worry is that P/CVE could detract from the trust which necessarily underlies the mental health professional's standing in relation to the individuals, families, and communities they serve. Moreover, governments could misuse P/CVE to target opposition groups or detain human rights activists.

*Lack of capacity* means that P/CVE programs cannot expect mental health service organizations to carry additional burdens without ensuring that they have the organizational capacity to do so, which is in part resource dependent.

## A possible path forward for violence prevention

Many low- and middle-income countries face serious persistent problems with violence of several types, including but certainly not limited to, violent extremism and hate-motivated violence. These have major negative impacts on public safety and public mental health and physical health. Preventing these types of violence cannot be achieved by law enforcement alone, and must involve broader civil society approaches, including public health strategies, which would include mental health.

The urgency of the threats demands that something be done, even though there is not yet adequate scientific evidence about the causes of violence or effective prevention practices or programs. However, efforts to prevent violence should not cause additional harm or make matters worse, which unfortunately can occur when policies or programs are driven by fear.

Over the past decade, GMH has established core commitments to utilizing evidence-based practices, addressing the treatment gap, combating stigma, and building local capacity, but has not yet achieved much with regard to violence prevention. P/CVE, which is an even more recent field, has little to no evidence-based practices, has been trying to make inroads with mental health professionals, and has been accused of increasing stigma to individuals and communities. This could mean that GMH could be in a position to help P/CVE evolve into a field with greater scientific legitimacy and community acceptance. In doing so, GMH could also enhance its own capacity for violence prevention.

GMH could best assist P/CVE by building on its aforementioned core commitments. This should entail strengthening civil society leadership of programs (including but not limited to mental health professional leadership), strengthening community collaboration, and building evidence of effective programs. It should also assist P/CVE in moving away from focus on a single faith and instead be based upon legitimate evidence of current threats. More than that, any new initiatives should not only avoid security terminology but should not be bound by current P/CVE frameworks. The goal of new initiatives should be to address a broader range of perceived threats to communities and individuals. Initiatives should also draw upon a range of strategies including building individual and community resilience, promoting youth development, and reducing involvement in all forms of violence. They should seek to add mental health resources to communities, which could also help to mitigate community concerns.

Given the concerns about discrimination and stigmatization, all activities should not intentionally or unintentionally give in to bias or discrimination against any community. Rather, a core value and practice should be to actively oppose discrimination and to stand up for the civil rights and liberties of patients, families, and communities. This could be done, for example, through strict protocols for the sharing of information gathered through clinical contacts or community engagement. Similarly, programs must take care to not compromise the ethics or standards of professional practice, even for public safety.

Violence prevention is probably best not presented as a stand-alone program or activity. Instead, initiatives should seek to integrate violence prevention with existing public health and mental health programs and priorities. Also, initiatives should not plan for new violence prevention programs involving mental health professionals or psychosocial workers without ensuring that service organizations have adequate capacity to carry them out on top of their other obligations. Instead, it is best make sure that programs have the capacity to be impactful and sustainable.

Lastly, additional research is needed to address many key questions concerning P/CVE and targeted violence prevention, which would also include questions pertaining to GMH. One set of questions pertains to the possible overlap between mental health problems and violent radicalization or other forms of targeted violence, such as: Are mental illness and personality disorders associated with a higher risk of being radicalized to a terrorist ideology? A second set of questions pertains to the key roles played by intimates and trusted gatekeepers in mitigating the pathways to violent and hateful ideologies, as well as other forms of targeted violence: What factors would facilitate or impede them from reporting to law enforcement or other helpers when someone they know could be a danger to others? A third set of questions pertain to risk assessment: Can we develop tools which can be used by community practitioners and that can reliably assess a person's risk for targeted violence? Last but not least, a fourth set of questions pertains to the evaluation of programs for primary, secondary, or tertiary prevention: What programs and practices effectively diminish individual and community risks for targeted violence?

## Conclusion

GMH should be open to addressing the challenges of violent extremism in low- and middle-income countries, but it should do so based upon existing mental health and public health values, practices, and evidence. GMH could help by critically appraising P/CVE practices and by working with multidisciplinary stakeholders to develop new evidence-based and best practice models that are rooted in civil society ownership, community collaboration, broader prevention programing, and non-securitized approaches.
